# Tungiasis-related life quality impairment in children living in rural Kenya

**DOI:** 10.1371/journal.pntd.0005939

**Published:** 2018-01-08

**Authors:** Susanne Wiese, Lynne Elson, Hermann Feldmeier

**Affiliations:** 1 Institute of Microbiology and Hygiene, University Medicine Berlin, Germany; 2 WAJIMIDA Jigger Campaign, Dabaso Tujengane CBO, Watamu, Kenya; Hitit University, Faculty of Medicine, TURKEY

## Abstract

**Background:**

Tungiasis (sand flea disease) is a neglected tropical skin disease caused by female sand fleas (*Tunga spp*.) embedded in the skin of the host. The disease is common in sub-Saharan Africa and predominantly affects children living in impoverished rural communities. In these settings tungiasis is associated with important morbidity. Whether tungiasis impairs life quality has never been studied.

**Methods:**

The study was performed in 50 children with tungiasis, living in resource-poor communities in coastal Kenya. Based on the Dermatology Life Quality Index (DLQI) a tool was developed to determine life quality impairment associated with tungiasis in children, the tungiasis-related Dermatology of Life Quality Index (tungiasis-related-DLQI). Pain and itching were assessed using visual scales ranging from 0–3 points. The intensity of infection and the acute and chronic severity of tungiasis were determined using standard methods.

**Results:**

Seventy eight percent of the patients reported a moderate to very large effect of tungiasis on life quality at the time of the diagnosis. The degree of impairment correlated with the number of viable sand fleas present in the skin (rho = 0.64, p < 0.001), the severity score of acute clinical pathology (rho = 0.74, p < 0.001), and the intensity of pain (rho = 0.82, p < 0.001). Disturbance of sleep and concentration difficulties were the most frequent restriction categories (86% and 84%, respectively). Four weeks after curative treatment, life quality had improved significantly. On the individual level the amelioration of life quality correlated closely with the regression of clinical pathology (rho = 0.61, p < 0.001).

**Conclusion:**

The parasitic skin disease tungiasis considerably impairs life quality in children in rural Kenya. After effective treatment, life quality improves rapidly.

## Author summary

Although tungiasis (sand flea disease) is associated with important morbidity and affects millions of people in South America, the Caribbean and sub-Saharan Africa, it has been largely ignored by health care providers up to now. In this study we show that the restriction of life quality due to tungiasis goes far beyond physical aspects and that the disease is also a mental and emotional strain on affected children in rural Kenya. Almost eighty percent of the diseased individuals reported a moderate to very large effect of tungiasis on their life quality. The degree of perceived life quality impairment correlated with the number of embedded sand fleas and pain. Sleep disturbance and concentration difficulties were the most frequent impairment. Effective treatment led to a rapid improvement of pathology and, subsequently, to an improved quality of life. The results of this study give substantial evidence that tungiasis is an important health hazard deserving more attention from policy makers and international donors.

## Introduction

Tungiasis (sand flea disease) is a parasitic skin disease caused by female sand fleas (*Tunga spp*.*)* penetrated into the skin of humans or animals [1]. It belongs to the ever-growing group of neglected tropical diseases (NTDs) which are infectious diseases prevalent in the tropical and subtropical regions and characterized by affecting the health of the world's poorest people and limiting their productivity. Tungiasis is particularly neglected in the sense that hitherto little research has been carried out with regard to disease burden and that health care providers in endemic areas commonly ignore the condition. Sand flea disease predominantly affects people living in poverty: in shanty towns at the periphery of metropolitan areas, in the rural hinterland or in isolated communities at the coast in South America, the Caribbean and sub-Saharan Africa including Madagascar [2–9]. Children and the elderly bear the highest disease burden [5,6,10]. In these population groups prevalence may be as high as 65%. Children frequently carry dozens of embedded sand fleas simultaneously [5,10].

In endemic areas 95 to 98% of all tungiasis lesions occur at the feet [10,11]. The toes, the sole and the heel are typical predilection sites [12]. Once embedded in the skin, the female sand flea undergoes a massive hypertrophy, and within two weeks reaches the size of a pea. Through an opening of about 250 μm the parasite remains in contact with the environment [13]. Being a continuously enlarging and biologically active foreign body located in the epidermis, embedded sand fleas cause an intense inflammatory response [12,14,15]. Bacterial superinfection is common and intensifies the inflammation [16]. Intense pain and itching are almost constant [13]. Frequent sequels are suppuration, ulcers, deep fissures, periungual oedema as well as deformation of nails and toes [12,14]. Although an effective and safe treatment exists it is not yet available in endemic countries [17–19]. Therefore, embedded sand fleas are removed by inappropriate sharp and non-sterile instruments such as needles, safety pins or razor blades [10,19]. This further increases the risk of bacterial superinfection and intense inflammation. Constant re-infection–as is the rule in endemic settings—impairs mobility, eventually leads to mutilation of the feet and immobilization of the patient. Anecdotal observations suggest that the restricted mobility may have a detrimental effect on household economics and impair school performance in children, mainly due to high absenteeism [19].

It is reasonable to assume that tungiasis causes mental strain and distress. In a setting where people rarely wear closed shoes the disease cannot be hidden in public and, since it is associated with poverty, it stigmatizes its victims [19]. In school, children are teased and ridiculed.

Whether tungiasis has an impact on life quality has never been investigated in a systematic manner. This study, therefore, aimed at assessing life quality in children living in a tungiasis-endemic area in rural Kenya.

## Materials and methods

### Study area and population

The study was performed in Kakuyuni Sublocation, Kilifi County, coastal Kenya, from September to October 2014. In the area, tungiasis is endemic with prevalences ranging from 30 to 85% in school-age children as determined in a school survey prior to the study (S1 Appendix).

Demographic data were collected within the larger survey addressing the epidemiology of tungiasis in the area. In Kilifi county communities are small and consist of two to five homesteads—clusters of houses—which are located about 100 m from each other, separated by fields or bushland. Usually four to six children sleep together with their parents in the same room, frequently on rugs put on the floor, more seldom on a dilapidated mattress, and rarely in a bed. Eighty five percent of the rooms do not have a solid floor, facilitating the propagation of the parasite inside the house. Eighty eight percent of the households have domestic animals such as goats, chicken, cats and dogs. In 89% of the homesteads the income is less than the official minimum wage of US$ 2 per day and thus fall in the lowest income bracket. School- age children very rarely own shoes and make their long way to school barefooted [20].

### Study design

Community health workers were asked to identify households with tungiasis or to find out where they knew tungiasis had occurred previously. Children aged 5 to 14 years with at least six tungiasis lesions (viable, non-viable or manipulated) were eligible for the study.

A total of 50 patients–between 2 and 15 children in each case—were recruited from five villages. In order to avoid family-related inclusion bias only the first child in a household identified to have tungiasis was eligible for the study.

The intensity of infection (number of embedded sand fleas) and severity of tungiasis was determined by standardized procedures [5,21]. Since in endemic areas female sandfleas almost constantly penetrate the skin of the feet, the examination was limited to this topographic site [10,11].

Eligible patients were explained the procedure and a caregiver (usually the mother) was asked for informed written consent. The feet of the patient were carefully washed with soap in a bucket. Thereafter, the feet were thoroughly examined by the principal investigator (SW) in a room in which the privacy of the patient was guaranteed. Lesions were staged according to the Fortaleza classification and counted [13]:

- stage I: penetrating sand flea
- stage II: brownish/black dot with a diameter of 1–2 mm
- stage III: circular yellow-white watch glass-like patch with a diameter of 3–10 mm and with a central black dot
- stage IV: brownish-black crust with or without surrounding necrosis

Stage I to III are viable sand fleas; in stage IV the parasite is dying or already dead [12,21].

Lesions manipulated with a sharp instrument, such as a needle, a safety pin, a thorn or a razor blade were documented as manipulated lesions. Patients were not asked who had tried to remove embedded sand fleas.

Clinical pathology was assessed semi-quantitatively, using previously established severity scores for acute and chronic tungiasis (SSAT; SSCT) [21]. The SSAT varies from 0–30 points, the SSCT from 0–32 points. Pain and itching were assessed using visual scales ranging from 0–3 points (S2 Appendix). Deliberately, the figures were kept very simple to make them understandable even for small children with little school education.

### Tungiasis-specific Dermatology Life Quality Index (DLQI)

The DLQI is a simple tool widely used to determine skin-associated life quality impairment. The English original was developed by Finlay and Khan [22], and is available at http://www.cardiff.ac.uk/dermatology/quality-of-life/. The DLQI is validated for an array of skin diseases of infectious and non-infectious origin [23,24].

In a first step, we modified the original questionnaire such that the tool measures the characteristic sequels of an inflammatory parasitic skin disease located at the feet. Next, we adapted the wording of the questions to the vocabulary and attitudes of Kenyan children. This resulted in a tungiasis-related DLQI with six categories of impairment and a score ranging from 0 to 18 points (S3 Appendix). Categories of impairment are as follows: feeling of shame, impairment of leisure activities, difficulty in walking, impairment of concentration during classes, social exclusion and sleep disturbances. The last four of these six categories were assessed using visual analogue scales (S2 Appendix), the first two just verbally.

The tungiasis-related DLQI was translated into Swahili, pre-tested in children, back-translated into English, then refined and translated into Swahili again. The questions were read out loudly and explained to the patients in a standardized manner by one of the native Swahili-speaking community health workers.

Children were shown the visual scales, depicting the categories of impairment one by one and were asked to point to the corresponding figure with their fingers. Answers were categorized as follows: no restriction perceived = 0, only a small restriction perceived = 1 point, important restriction perceived = 2 points, severe restriction perceived = 3 points. The points for each category were added up to form the tungiasis-related DLQI (Table 1).

**Table 1:** Interpretation of the tungiasis-related DLQI scores.

Scores	Assumed impact on patient’s life quality
0–1	None
2–3	Small
4–8	Moderate
9–13	Large
14–18	Very large

Immediately after examination and interview the patients were referred to the local health centre for treatment. There they were treated according to national guidelines (http://www.jigger-ahadi.org/National Policy Guidelines for Prevention and Control.pdf; accessed November 29, 2016).

Patients were asked to present themselves at a determined location, usually the school, four weeks after treatment for follow up.

### Statistical analysis

The data were entered into an Excel database (Excel Version 2013, Microsoft, Redmont, Washington, USA) and checked for errors which might have occurred during data entry. The data analysis was carried out using the Analysis ToolPack Add-In (Microsoft, Redmont, Washington, USA). The median and the interquartile range were calculated as indicators of central tendency and dispersion of the data, respectively. Since the data did not follow a normal distribution, non-parametric tests were used. The Mann-Whitney-U test was used to compare the modified Dermatology Life Quality Index (mDLQI) between subgroups of patients, and the Wilcoxon matched pairs signed rank test for the comparison of variables before and after treatment. The Spearman rank correlation coefficient was calculated to determine the significance of correlations. Relative frequencies were compared with the Chi-squared and Fisher exact tests.

The sample size estimate was based on the assumption of a 25% difference in tungiasis-related life quality impairment before and after treatment. Estimating a dropout rate of 5%, 45 patients were needed for complete data analysis (probability = 0.95; power of the test = 0.80).

### Ethical considerations

The study was approved by the Ethics Review Committee at Pwani University, Kilifi County; approval number ERC/PhD/010/2014. The custodians and their protégés were informed about the objectives and procedures of the study in Swahili. The right to deny participation and withdraw consent at any given time was clearly explained.

The informed consent form was read out loud word by word in Swahili and explained further when required; questions of the custodian and the children were discussed and answered by a community health worker. Informed assent was obtained from the patients before examination and treatment. Written consent was obtained via fingerprint or signature from the legal guardian or the headmaster of the school in which the patient was enrolled. Participants were only examined in the presence of their mother or the headmaster.

For other illnesses requiring treatment a referral form was prepared by a community health worker, and patients were referred to the Health Facility in Kakuyuni. Treatment was also made available for household members with tungiasis who did not participate in the study.

## Results

Fifty patients were included in the study, 35 of them male and 15 female. The demographic and clinical characteristics are summarized in Table 2. The median age was 8 years (range 5–14). Fifty four percent of the patients had more than six viable lesions (boys 57%, girls 47%). The maximum number of lesions was 457; 162 of them viable. Severity scores for acute and chronic tungiasis were: median SSAT 10 (maximum 27) and median SSCT 6 (maximum SSCT 11), respectively. Of the total 3,556 lesions present at baseline 58% had been manipulated by the patient or the caregiver. All participants showed at least one manipulated lesion, the median number being 33 (maximum 196). Overall, the intensity of tungiasis and the severity of disease was higher in boys than in girls.

**Table 2:** Demographic and clinical characteristics of children with tungiasis (n = 50).

**Characteristic**		**N (%)**	
	**total**	**Male**	**Female**
Sex	50 (100)	35 (70)	15 (30)
Age (years)			
Median	8	9	8
Range	5–14	5–14	5–14
**Clinical pathology**	**N (%)**		
Erythema/warmness/oedema	43 (86)	29 (83)	14 (93)
Suppuration/ulcer/abscess	27 (54)	21 (60)	6 (40)
Local pain (points)*			
0	6 (12)	4 (11)	2 (13)
1	19 (38)	11 (31)	8 (53)
2	15 (30)	11 (31)	4 (27)
3	10 (20)	9 (26)	1 (7)
Itching (points)*			
0	3 (6)	2 (6)	1 (7)
1	32 (64)	22 (63)	10 (67)
2	9 (18)	6 (17)	3 (20)
3	6 (12)	5 (14)	1 (7)
**Number of lesions**	**Median (IQR)**		
All lesions types	52.5 (40–81.5)	59 (41.5–85.5)	43 (27–75)
Viable lesions	7 (3–12.75)	8 (4–15.5)	4 (1–7.5)
Non-viable lesions	12.5 (6–19.8)	13 (6.5–13)	9 (4.5–18.5)
Manipulated lesions	33 (25–48.75)	37 (26–50)	31 (14–48)
**Severity scores**	**Median (IQR)**		
SSAT	10 (7.25–12)	10 (8–14.5)	8 (7–10.5)
SSCT	6 (4–7.5)	6.5 (4–8)	6 (4.5–6)

* indicated by the patient using a visual scale; see Material and methods

IQR: interquartile range

SSAT: severity score for acute tungiasis

SSCT: severity score for chronic tungiasis

**Fig 1:**
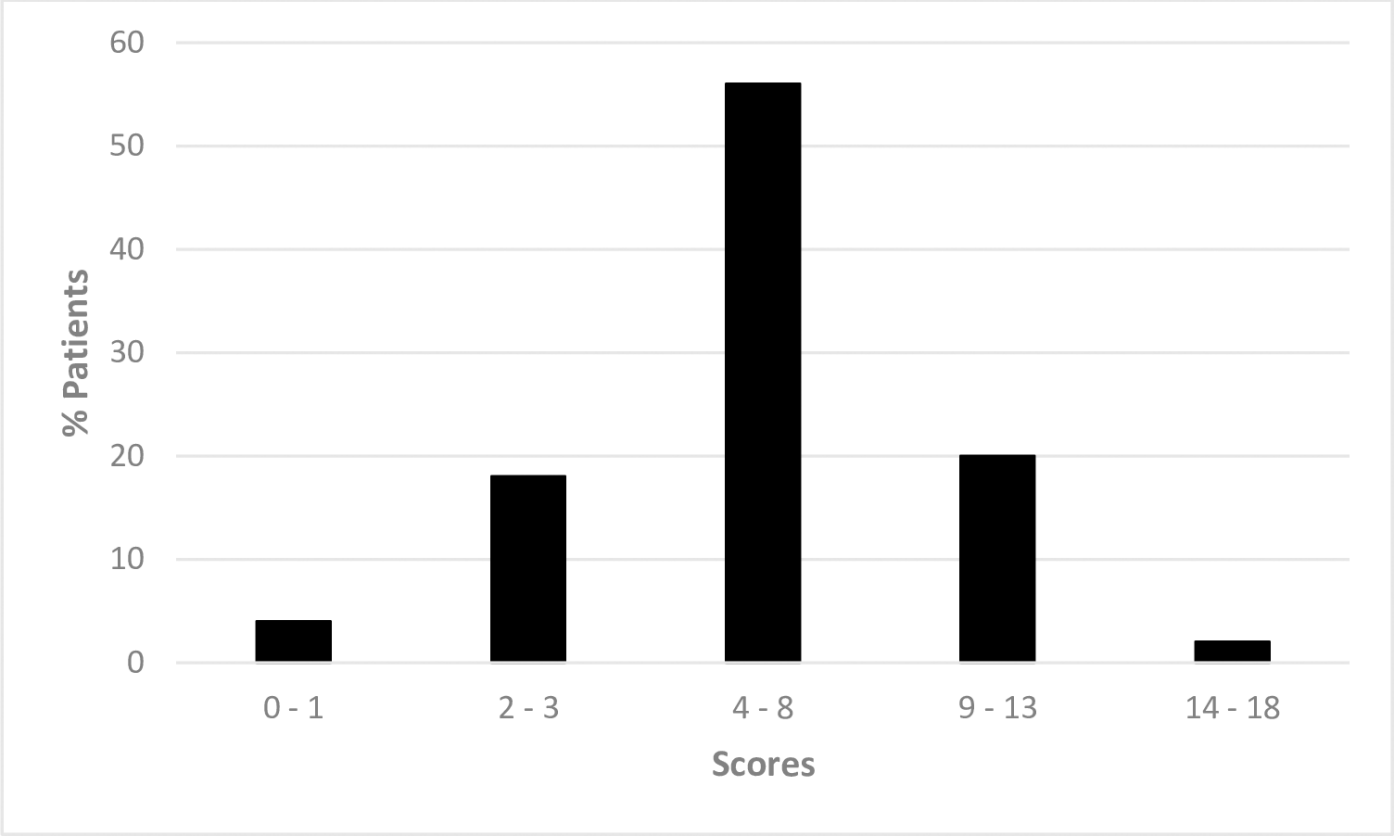
Distribution of the tungiasis-related DLQI scores (n = 50).

The tungiasis-related DLQI showed that 78% of the patients reported a moderate to a very large restriction of their life quality (Table 3). The majority of the patients (56%, Fig 1) showed a moderate impairment corresponding to a median tungiasis-related DLQI of 6 points (25^th^ and 75^th^ percentile 4–8.5 for boys and 3.5–8 for girls, respectively). Sleep disturbance and concentration difficulties in class were the impairment categories most commonly reported (Table 4). None of the restriction categories differed between boys and girls.

**Table 3:** Tungiasis-related life quality impairment (n = 50).

Tungiasis-related DLQI-scores	Impact on life quality	N (%) Total	N (%) male	N (%) female	P-value (♂ versus ♀)
(0–1 points)	No effect	2 (4)	1 (3)	1 (7)	0.51
(2–3 points)	Small effect	9 (18)	6 (17)	3 (20)	1.00
(4–8 points)	Moderate effect	28 (56)	19 (54)	9 (60)	0.76
(9–13 points)	Large effect	10 (20)	8 (23)	2 (13)	0.70
(14–18 points)	Very large effect	1 (2)	1 (3)	0 (0)	1.00

**Table 4:** Restriction categories in patients with tungiasis (n = 50).

Restriction category	N (%) Total	N (%) male	N (%) female	P value (♂ versus ♀)
Sleep disturbances	43 (86)	32 (91)	11 (73)	0.18
Concentration difficulty	42 (84)	30 (86)	12 (80)	0.68
Feeling of shame	38 (76)	28 (80)	10 (67)	0.47
Restriction of leisure activities	38 (76)	28 (80)	10 (67)	0.47
Walking difficulty	37 (74)	26 (74)	11 (73)	1.00
Social exclusion	31 (62)	21 (60)	10 (67)	0.76
**Tungiasis-related DLQI score**				
Median (IQR)	6 (4–8)	6 (4–8.5)	6 (3.5–8)	0.41

**Fig 2:**
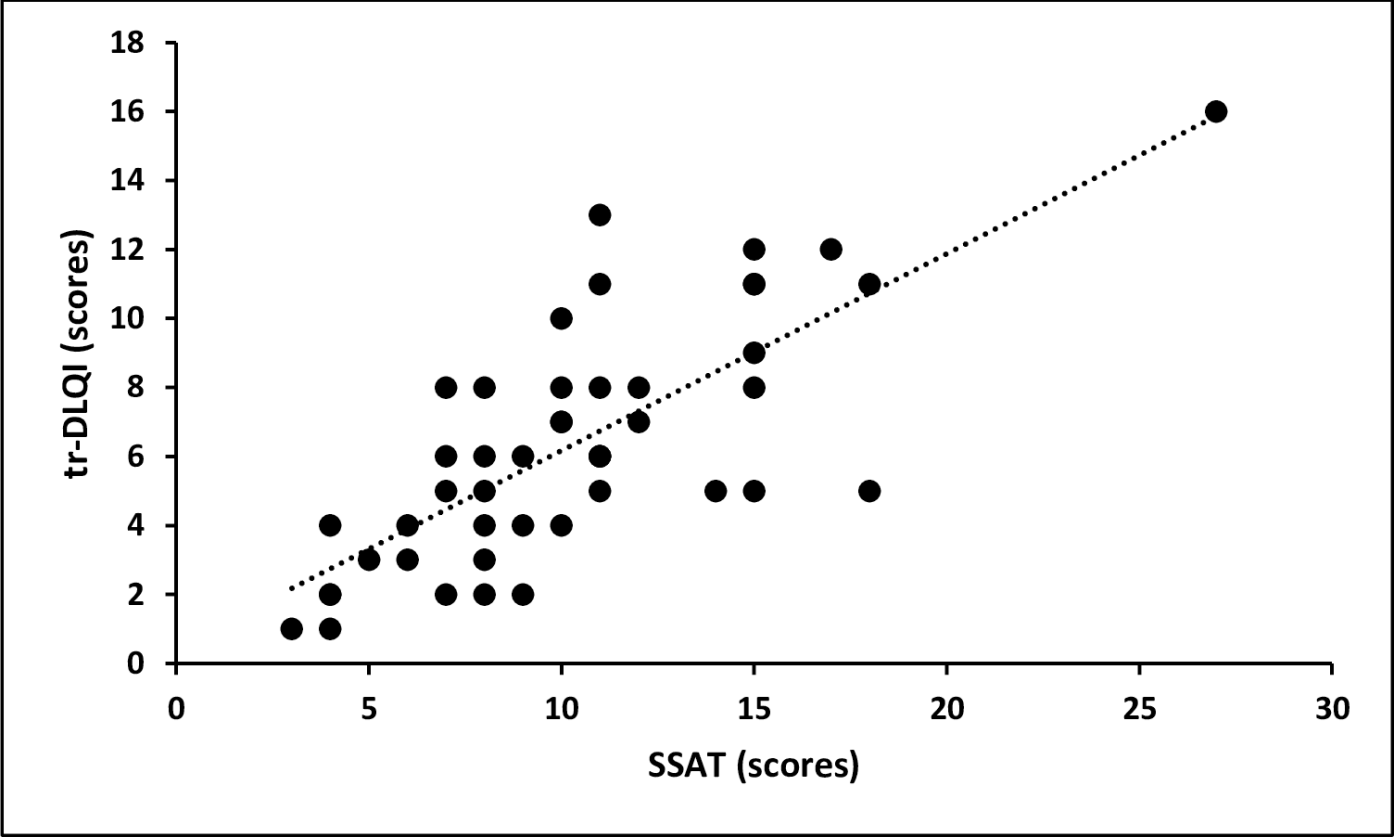
Correlation between severity of tungiasis measured by SSAT and the tungiasis-related DLQI (rho = 0.74, p < 0.001); dotted line = regression curve.

There was a strong correlation between the severity of the acute clinical pathology as measured by the SSAT and the impairment of the life quality (rho = 0.74, p < 0.001, Fig 2). The intensity of pain (as assessed by the visual scale) showed an even stronger correlation with the tungiasis-related DLQI (rho = 0.82, p < 0.001). The intensity of itching was less strongly correlated (rho = 0.61, p < 0.001). There was only a weak correlation between tungiasis-associated chronic pathology as measured by the SSCT and the tungiasis-related DLQI (rho = 0.27, p = 0.06). Whereas the total number of lesions and the number of manipulated lesions did not correlate with the tungiasis-related DLQI (rho = 0.23, p = 0.10 and rho = 0.004, p = 0.98, respectively), the number of viable lesions did (rho = 0.64, p < 0.001).

**Fig 3:**
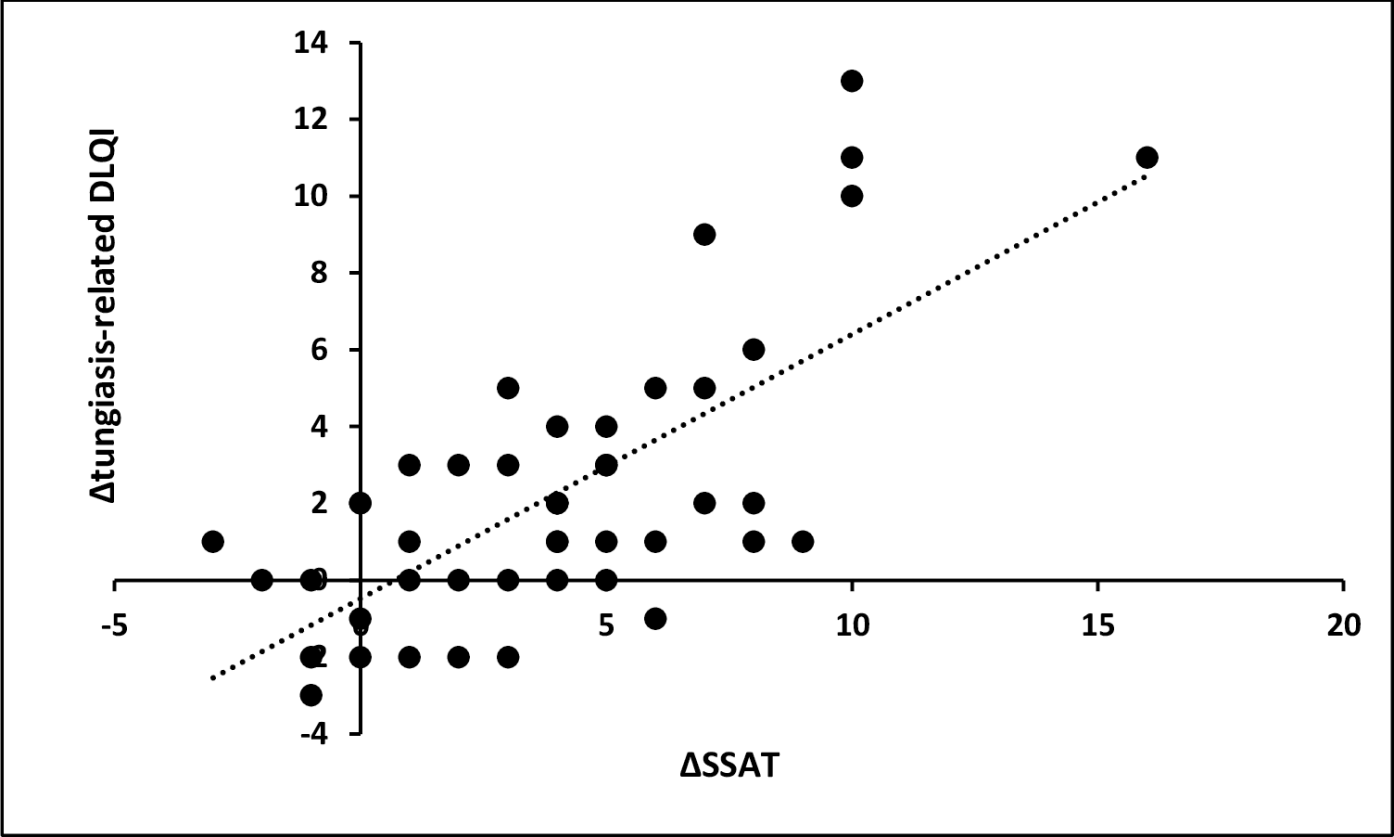
Correlation between individual decrease in severity of acute tungiasis (Δ SSAT) and decrease in impairment of life quality (Δ tungiasis-related DLQI) after treatment (rho = 0.61, p < 0.001); dotted line = regression curve.

Of the 50 patients, 46 presented again four weeks after treatment. In these patients acute clinical pathology had decreased significantly: median SSAT at baseline = 10 (interquartile range 7.25–12) versus 7 at follow up (interquartile range 3.25–8; p<0.001). This was accompanied by a slight though significant decrease of the tungiasis-related -DLQI: median = 6 (interquartile range 4–8) versus 5 (interquartile range 1.25–6; p<0.001, Table 5). The patients noted a clear amelioration in the restriction categories; concentration difficulty in class and impairment of leisure activities (p = 0.001 and p < 0.001, respectively) as well as feeling of shame and walking difficulties (p = 0.007 and p = 0.003). On the individual level there was a highly significant correlation between the reduction of acute clinical pathology and the amelioration of life quality (rho = 0.61, p < 0.001; Fig 3).

**Table 5:** Improvement of clinical pathology and of life quality four weeks after treatment (n = 46).

Category of impairment	N (%) before treatment	N (%) after treatment	P value
Sleep disturbances	39 (85)	34 (74)	0.332
Concentration difficulty	39 (85)	25 (54)	0.001
Feeling of shame	34 (74)	25 (54)	0.007
Restriction of leisure activities	35 (76)	23 (50)	<0.001
Walking difficulty	34 (74)	25 (54)	0.003
Social exclusion	29 (63)	24 (52)	0.087
**tr-DLQI scores**			
Median (IQR)	6 (4–8)	5 (1.25–6)	<0.001
**SSAT score**			
Median (IQR)	10 (7.25–12)	7 (3.25–8)	<0.001

## Discussion

The group of 50 tungiasis patients enrolled in the study exhibited a broad spectrum of tungiasis-associated pathology, from moderate to severe disease. The fact that all children had manipulated lesions emphasizes that the embedded fleas were causing suffering, and in an act of despair, either they themselves or their caregivers tried to relieve that suffering by physical removal of the flea. In fact, 96% of the patients perceived their life quality to be impaired and 78% considered the impairment moderate to severe according to our mDLQI. This frequency of life quality impairment is similar to those reported from studies in patients with other neglected tropical parasitic skin diseases such as scabies, hookworm-related cutaneous larva migrans and cutaneous leishmaniasis [25–27]. However, patients with these other skin diseases classified the impairment as severe less frequently than patients with tungiasis.

Other than social exclusion, all impairment categories were reported in similar frequencies (range 62 to 86%) to each other. Sleep disturbances due to pain and itching were perceived as particularly impairing. This is plausible, because itching usually intensifies at night, and pain is perceived more intensive in a quiet environment when a person tries to fall asleep. A lack of sufficient and re-generative sleep leads to tiredness, bad mood and concentration difficulties in class, another impairment category noted by the patients. In the long term, sleep disorders might cause the development of psychological problems such as anxiety [28].

To alleviate the pain patients avoid placing the whole foot on the ground while walking leading to a classical gait which is readily recognized as a tungiasis patient from a long distance [29]. Obviously, impaired mobility limits the typical leisure activities of children in rural Africa.

Since children with tungiasis are ridiculed at school and because it is widely known that tungiasis affects the poorest of the poor, it is understandable that the feeling of shame and stigmatization are perceived as important restrictions. Probably, both restriction categories are also interlinked with social exclusion. Since the skin alterations are located on visible body parts, they are difficult to conceal and, on the long run, may lead to withdrawal and/or exclusion from society. Leprosy is paradigmatical for such sequels [30]. Patients may be confronted with ignorance or misconceptions regarding the aetiology of their skin disease, such as the fear that the condition is contagious or caused by poor personal hygiene–assumptions eventually leading to stigmatization [31,32]. This is the case, for instance, in lymphatic filariasis, a parasitic skin disease leading to gross lymphoedema of legs, arms and the genitals [33,34]. Stigmatization is detrimental to the well-being of the patient, causing distress and potentially inducing mental disorder [32,35]. We did not find any significant difference in impairment quality and frequency between boys and girls. A similar observation was made by Schuster et al. in patients with hookworm-related cutaneous larva migrans living in a slum in Manaus, Brazil [25].

The importance of this study is multifold. For the first time, it was shown that tungiasis—a parasitic skin disease still considered to be a nuisance rather than an important parasitic disease in standard text books–impairs life quality. The impairment categories perceived by the patients do not only reflect the clinical pathology caused by the embedded parasite (such as itching, pain and restricted mobility) but also confirm the mental strain and distress it causes. Since the disease cannot be concealed in the setting where the patients live, children are exposed to ridicule and stigmatization. Besides, the impaired mobility is presumably responsible for restricted social interactions and hinders typical leisure activities of children living in rural Africa.

Second, the study clearly shows that a cause-effect-relationship exists between tungiasis and impaired life quality. At baseline the intensity and severity of tungiasis as well as the degree of pain were all positively correlated to the degree of life quality impairment and the degree of association was strong. After effective treatment life quality ameliorated or was restored. On the individual level there was a highly significant correlation between the reduction of disease severity and the amelioration of life quality after treatment. Finally, life quality was not restored in the patients who became re-infected during the observation period (Fig 3). Whereas in previous studies on life quality in neglected parasitic skin diseases it remained unknown to which degree the setting (such as living in poverty) contributed to the impairment categories perceived by the patients, this study convincingly shows that it is the disease—and not the setting—which causes an important impairment of life quality in children.

Third, the results of this study are a strong argument that tungiasis is an important health hazard—on the individual level as well as on the public health level—and that health care providers and regulators should give it the priority it deserves. Effective treatment and prevention do exist and should be made accessible for all patients in endemic areas [17,36].

The study has a couple of limitations: First, an observation time of four weeks is too short to completely reverse clinical pathology and, hence, it is also too short to determine whether life quality will be completely restored after all lesions have healed. However, a prolongation of the follow up period would have increased the risk of re-infection. In this case, it would have been impossible to distinguish between inflammation still persisting from an original infection and clinical pathology resulting from newly penetrated sand fleas. Second, a higher power of the study would have been preferable. However, due to financial and logistic constraints, it was impossible to increase the study size and to include a control group. Third, there is an over-representation of males in the study population. This sex imbalance reflecting the higher prevalence of tungiasis in males in the endemic areas [4, 5] might have been caused by a selection bias.

Taken together, a simple tool enabled the demonstration of a cause-effect-relationship between the presence of a wholly neglected tropical disease and impaired life quality in children living in an impoverished setting in rural Africa. This work highlights the urgent need for international donors to support the development and registration of curative and preventive interventions, and for policy makers and health officials in endemic countries to address tungiasis to avoid this suffering.

## Supporting information

S1 Appendix Database. Prevalence of tungiasis in 5 schools in Kilifi County.
(DOCX)

S2 Appendix Visual analogue scales. Not at all = 0 points, Only a little = 1 point, Quite a lot = 2 points, Very much = 3 points.
(DOCX)

S3 Appendix mDLQI for children tungiasis patients (5–15 y) back-translated into English from Swahili. Not at all = 0 points, Only a little = 1 point, Quite a lot = 2 points, Very much = 3 points.
(DOCX)
