# Supplementary material for: Tungiasis-related life quality impairment in children living in rural Kenya
Source: PLoS Negl Trop Dis. 2018 Jan 8;12(1):e0005939. doi: 10.1371/journal.pntd.0005939 (PMC5757912; doi:10.1371/journal.pntd.0005939)
Supplement: S2 Appendix — Not at all = 0 points, Only a little = 1 point, Quite a lot = 2 points, Very much = 3 points. (DOCX) [file pntd.0005939.s002.docx]

**Walking difficulties**

**
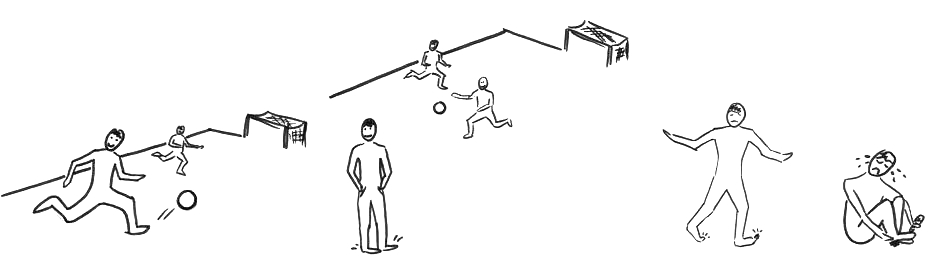
**

Not at all Only a little Quite a lot Very much

**Concentration difficulties**

**
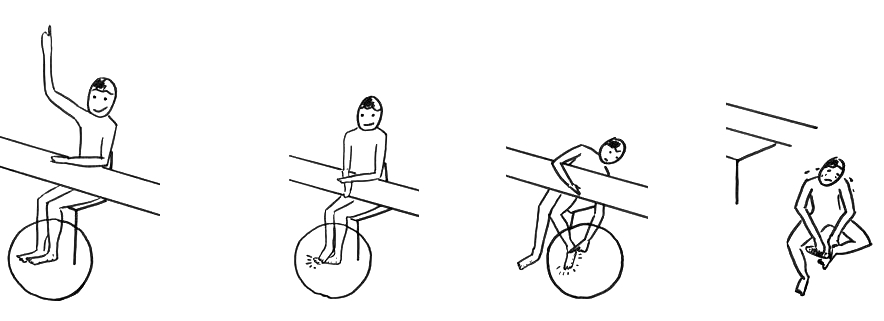
**

Not at all Only a little Quite a lot Very much

**Social exclusion**

**
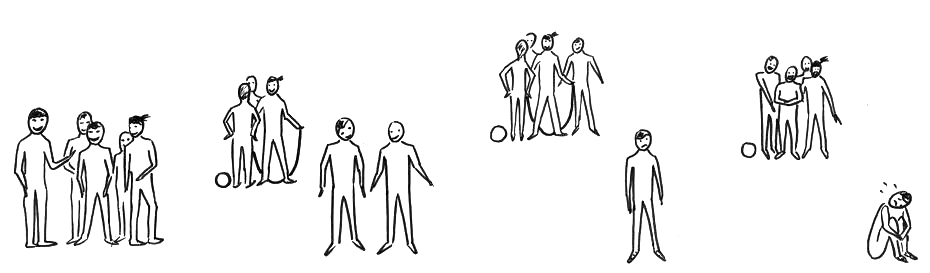
**

Not at all Only a little Quite a lot Very much

**Sleeping difficulties**


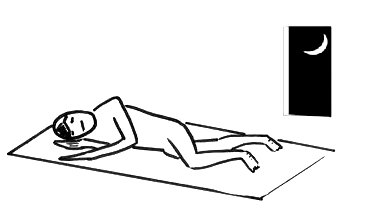


Not at all


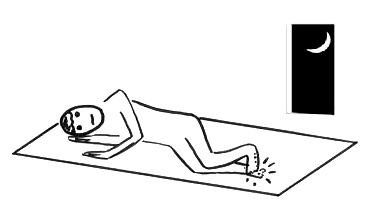


Only a little


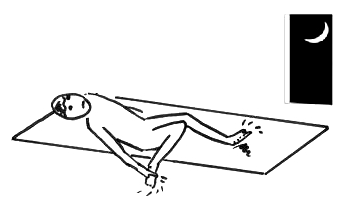


Quite a lot


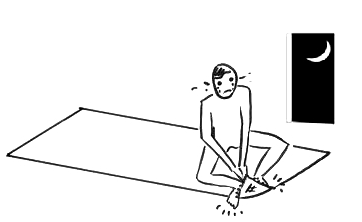


Very much

**Pain**


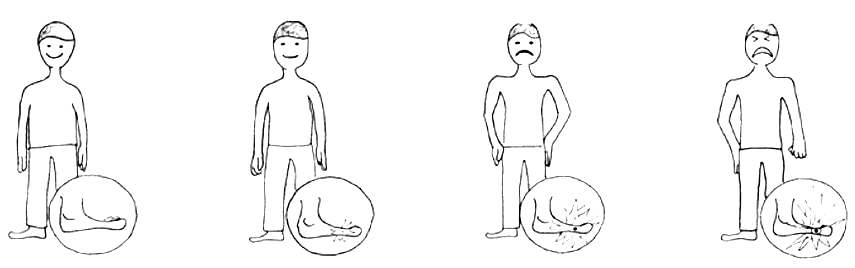


Not at all Only a little Quite a lot Very much

**Itching**

**
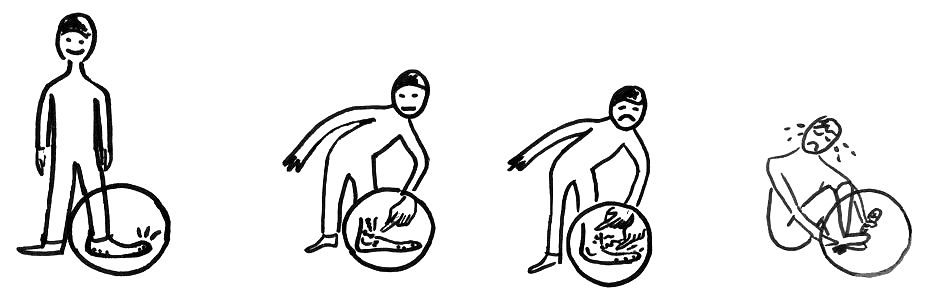
**

Not at all Only a little Quite a lot Very much
