# Supplementary material for: Tungiasis-related life quality impairment in children living in rural Kenya
Source: PLoS Negl Trop Dis. 2018 Jan 8;12(1):e0005939. doi: 10.1371/journal.pntd.0005939 (PMC5757912; doi:10.1371/journal.pntd.0005939)
Supplement: S3 Appendix — Not at all = 0 points, Only a little = 1 point, Quite a lot = 2 points, Very much = 3 points. (DOCX) [file pntd.0005939.s003.docx]

ID-no.__________ Date______/_____/ 2014 Village____________________________

Name_______________________________________________ Age________ Sex (m/f)___

1. During the last week, how **embarrassed** or **ashamed** did Not at all

you feel because of the jiggers? Only a little

Quite a lot

Very much

Note:_________________________________________________________________

1. During the last week, how much have the jiggers made it Not at all

difficult for you to **walk**? Only a little

Quite a lot Very much

Note:_________________________________________________________________

1. During the last week, how much did the jiggers Not at all

affect your **school work**? Only a little Quite a lot Very much

Note:_________________________________________________________________

1. During the last week, how much did the jiggers Not at all

affect your **leisure activities**? Only a little Quite a lot Very much

Note:_________________________________________________________________

1. During the last week, how much did the jiggers Not at all

affect your **friendships**? Only a little Quite a lot Very much

Note:_________________________________________________________________

1. During the last week, how much did the jiggers Not at all

affect your **sleep**? Only a little Quite a lot Very much

Note:_______________________________________________________________________
